# Cumulative Burden of Lifestyle Risk Factors on Cancer in Older Korean Men: A Nationwide Retrospective Cohort Study

**DOI:** 10.3390/cancers17030426

**Published:** 2025-01-27

**Authors:** Hee Joon Choi, Seung Hee Kim, Ye-Jee Kim, Young Sik Kim, Jung Hwan Kim, Min Kyu Sung, Seo Young Kang

**Affiliations:** 1 Eulji University School of Medicine, Daejeon 34824, Republic of Korea; 2 Department of Family Medicine, Wonkwang University Sanbon Hospital, Wonkwang University School of Medicine, Gunpo 15865, Republic of Korea; 3 Department of Clinical Epidemiology and Biostatistics, Asan Medical Center, University of Ulsan College of Medicine, Seoul 05505, Republic of Korea; 4 Department of Family Medicine, Asan Medical Center, University of Ulsan College of Medicine, Seoul 05505, Republic of Korea; 5 Department of Family Medicine, Gangnam Eulji Medical Center, Seoul 06047, Republic of Korea; 6 Division of Hepatobiliary and Pancreatic Surgery, Department of Surgery, Asan Medical Center, University of Ulsan College of Medicine, 88, Olympic-ro 43-gil, Songpa-gu, Seoul 05505, Republic of Korea; 7 Department of Family Medicine, Uijeongbu Eulji Medical Center, Eulji University School of Medicine, 712, Dongil-ro, Uijeongbu-si 11759, Republic of Korea

**Keywords:** lifestyle risk factors, cancer, South Korea, older Korean men, smoking, alcohol consumption, physical activity

## Abstract

Background/Objectives: Cancer prevalence is increasing globally due to aging populations. Certain lifestyles are associated with cancer; however, the long-term combined effects of lifestyle factors on cancer has not been investigated. This study aimed to investigate the cumulative effect of lifestyle risk factors over 8 years on cancer in older Korean men. Methods: We assessed 64,756 men aged ≥65 years at the baseline year (2008–2009) using data from the National Health Insurance Service-Senior Cohort. Lifestyle factors, including smoking status, alcohol consumption, and physical activity, were evaluated over four consecutive phases (2002–2003, 2004–2005, 2006–2007, and 2008–2009) of health check-ups, and each participant’s overall lifestyle risk score was calculated after considering the value of a single poor lifestyle factor in each phase as 1, with a cumulative score ranging between 0 and 12. According to the lifestyle risk scores, the hazard ratios (HRs) and 95% confidence intervals (CIs) were calculated using Cox proportional hazard regression analysis. Results: Of the 64,756 men, 13,130 cases of cancer developed. The risk of cancer increased as the number of poor lifestyle factors and lifestyle risk score increased from the baseline year during the four phases. Compared to a score of 0–2, the HRs for cancer were 1.10 (0.98–1.23), 1.54 (1.37–1.73), and 1.72 (1.48–1.99) for scores of 3–5, 6–8, and 9–12, respectively. Conclusions: The risk of cancer increased as the cumulative burden of poor lifestyle habits over 8 years increased. Therefore, practicing and maintaining healthy lifestyles is crucial to preventing cancer in older Korean men.

## Simple Summary

Unhealthy lifestyles are known to increase the risk of cancer. This study investigated the long-term cumulative and combined effects of three major unhealthy lifestyle behaviors (smoking, drinking, and lack of regular physical activity) on cancer among older Korean men, after considering the high incidence of cancer and the dominance of correctable, unhealthy lifestyle behaviors among older men within South Korea. Overall, the risk of cancer increased along with increasing lifestyle risk scores reflecting unhealthy behaviors over an 8-year period. These findings highlight the importance of practicing and maintaining healthy lifestyles to prevent cancer in older Korean men.

## 1. Introduction

South Korea is facing an unprecedented increase in the number and percentage of older adults within its population given the country’s low birth rate and expanded life expectancy [1,2]. According to the “2024 Statics on the Ages” released by Statistics Korea, the elderly population in South Korea is projected to reach 9.938 million in 2024, representing 19.2% of the entire population [2]. Such a phenomenon places a burden upon many parts of society, one of which is the medical expenditure associated with the prevalence of non-communicable diseases in the elderly [3].

Cancer, a major type of non-communicable disease, is a leading cause of death in South Korea [4]. The “Causes of Death Statistics 2023” provided by Statistics Korea presents malignant neoplasm (cancer) as the number one cause of death in South Korea since 1983 [5]. According to the 2023 Korean Cancer Association Cancer Report, the incidence of cancer has consistently risen, and as of 2020, the total number of cancer cases was 247,952, which is a dramatic increase from the 144,896 cases reported in 2000 [6]. A study on the prediction of cancer incidence and mortality in Korea suggests 292,221 new cases and 83,770 deaths will occur in 2024 alone [7]. The severity of such a trend is likely to worsen considering the rapid aging of the South Korean population. For men aged 65 and above, the leading types of estimated new cases in 2024 were prostate cancer (15.8%), lung cancer (14.9%), colon and rectum cancer (13.5%), stomach cancer (10.7%), and liver cancer (6.7%) [7]. Such a trend is also observed globally, and a study on worldwide cancer statistics also determines population aging to be a leading cause of cancer [8].

Most cancers develop due to a complex combination of genetic, environmental, and lifestyle factors. Various lifestyle factors are known to strongly influence cancer risk, progression, and mortality [9]. Of all lifestyle factors, tobacco smoking is by far one of the most attributable factors of cancer that can be modified at an individual level [10]. It not only impairs the human respiratory system but also harmfully impacts most parts of the body [11]. A meta-analysis on the causal role of smoking in multiple diseases shows that genetic liability to smoking is associated with cancers of the lung, esophagus, head and neck, bladder, kidney, cervix, ovaries, and pancreas, and myeloid leukemia [12]. Alcohol consumption is another major lifestyle factor known to increase the risk of cancer. The International Agency for Research on Cancer classifies alcoholic beverages and the acetaldehyde produced from them as group 1 carcinogens, which is the most severe classification [13]. Common cancers associated with alcohol consumption include gastric, liver, esophageal, pharyngeal, laryngeal, and colorectal cancers [14]. Studies in Korea have also suggested that excess drinking increases the incidence of alcohol-related cancers and the risk of many others, including gastric, esophageal, and colorectal cancers. In addition, a lack of physical activity, sedentary behavior, and obesity are also known risks for cancer [9,15]. Epidemiological research has identified that maintaining physical activity can help reduce the risks of many cancers, claiming a strong association between the highest and lowest physical activity levels and a reduced risk of endometrial, colon, stomach, bladder, kidney, and esophageal cancers [16].

Research on the relationship between modifiable lifestyle factors (smoking, drinking, and physical activity) and cancer risk has been conducted, and the data suggest that a combination of these lifestyle factors can greatly affect the incidence of cancer. A study on colon cancer showed that when lifestyle factors such as alcohol intake, cigarette smoking, diet pattern, physical activity, and sleep were combined into a single lifestyle index, an increasing protective lifestyle factor index was associated with decreased risk of colon cancer [17]. Moreover, a meta-analysis showed that people in healthier lifestyle groups displayed a lower risk of incident cancer and cancer mortality overall [18]. While these studies have investigated the combined risk of lifestyle factors on cancer at a single point, there is no specific research that has focused on the long-term cumulative impact of lifestyle factors on cancer.

Additionally, previous studies have shown a distinct difference in lifestyle habits between sexes, with older Korean men having significantly unhealthier lifestyle behaviors than older Korean women [19,20]. Therefore, considering the noticeably high percentage of cancer incidence among the elderly and the dominance of correctable, unhealthy lifestyle behaviors among older men within South Korea, we aimed to investigate the cumulative and combined effects of unhealthy lifestyle behaviors on cancer in the Korean population. Specifically, this study evaluated the cumulative effect of 8 years of modifiable lifestyle factors, which were measured four times, on cancer development among older South Korean men.

## 2. Methods

### 2.1. Data Source and Study Population

We analyzed data from the National Health Insurance Service (NHIS)-Senior Cohort. In South Korea, the NHIS covers mandatory health insurance for approximately 97% of the population and provides health screenings to all insured members every consecutive year [21]. The NHIS-Senior Cohort, founded to support research on older adults, such as the analysis of risk factors and the prognosis of geriatric disease, consists of older adults ≥60 years from 2002. It includes participants in an anonymized format and contains information regarding their insurance premiums, history of hospital and clinic visits, health check-up results, information on nursing facilities, and use of long-term care services [22]. The institutional review board of the Asan Medical Center provided exemptive approval for this study (2022-0543).

**Figure 1:**
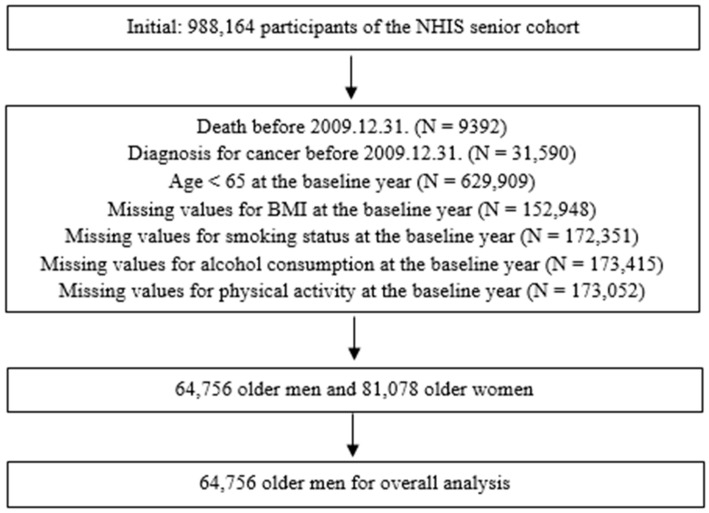
Flow diagram of the study population.

In this study, the baseline year was set at 2008–2009, and participants were followed up until 31 December 2019. Among the 988,164 participants in the NHIS-Senior Cohort, we excluded those who (1) died before the index date (31 December 2009; N = 9392); (2) were diagnosed with cancer before the index date (N = 31,590); (3) were aged <65 years at the baseline year (2008–2009; N = 629,909); and (4) had missing values for body mass index (BMI; N = 152,948), smoking status (N = 172,351), alcohol consumption (N = 173,415), and physical activity (N = 173,052) at the baseline year, leaving 64,756 older men and 81,078 older women. As this study was focused on older men, 64,756 participants were included in the analysis (Figure 1).

### 2.2. Evaluation of Lifestyle Factors

Lifestyle factors were evaluated biennially, and the factors assessed included smoking status, alcohol consumption, and regular exercise. Smoking status was classified as either current smoker or non-smoker, while alcohol consumption was categorized as either current drinker or non-drinker. Regarding regular exercise, participants reported their weekly exercise frequency. From 2002 to 2008, regular exercise was defined as exercising three or more times a week, whereas from 2009, it was defined as either involving 30 min of moderate exercise more than five times a week or performing a minimum of 20 min of intense exercise more than three times a week [23].

### 2.3. Definition of Lifestyle Risk Score

Three lifestyle factors—smoking, drinking, and physical activity—were evaluated four times over 8 years due to the national health screening taking place biennially in Korea. The degree of poor lifestyle was recorded as a lifestyle risk score. For each measurement, one point each was given for currently smoking, currently drinking, and not engaging in regular physical activity. Therefore, a combined score of 0–12 was assigned over the four evaluation periods. In this study, all three risk factors were given an equal weight when measuring cumulative risk, following previous studies that have weighed such factors equally [24,25].

### 2.4. Definition of Cancer

We assessed cancer based on the codes from the International Classification of Disease, 10th version (ICD-10). Participants diagnosed with and having received treatment for cancer at least twice were classified as patients with cancer (C00–C97).

### 2.5. Other Covariates

Covariates were evaluated from the baseline year 2008–2009. The sociodemographic variables included age, sex, BMI, and insurance premiums (medical aids, quartiles). In Korea, insurance premiums are calculated individually according to income and assets and therefore act as a major indicator of one’s socioeconomic status. The presence of hypertension, type 2 diabetes mellitus, and dyslipidemia was determined using ICD-10 codes. A participant was considered to have a comorbidity if insurance claims for outpatient or inpatient treatment were made at least three times with the relevant ICD-10 codes and the participant received medication for the comorbidity. The specific ICD-10 codes used were as follows: hypertension (I10, I11, I12, I13, I15), diabetes mellitus (E10, E11, E12, E13, E14), and dyslipidemia (E78).

### 2.6. Statistical Analysis

Participants were followed up from the index date to the date of diagnosis, death, or 31 December 2019, depending on which came first. Baseline characteristics were analyzed using descriptive statistics. Cumulative incidence rates (CIRs) of cancer were calculated according to lifestyle factors, and Cox proportional hazard regression analysis was used to evaluate the effect of lifestyle factors on cancer. Furthermore, the risk of cancer was evaluated based on the number of poor lifestyle factors at the baseline. We calculated the hazard ratio (HR) and 95% confidence interval (CI) for developing cancer when one, two, or three poor lifestyle factors were present in the baseline year compared to when no poor lifestyle factors were present. Then, the three lifestyle factors were assessed four times over an eight-year period (2002–2003, 2004–2005, 2006–2007, and 2008–2009) to create a lifestyle risk score and evaluate cancer incidence accordingly. For this, only 14,721 participants, out of 64,756 initial participants, were included in the analysis after excluding those with missing values for lifestyle factors during each measurement. The HRs and 95% CIs for lifestyle risk scores of 3–5, 6–8, and 9–12 compared to risk scores of 0–2 were calculated using Cox proportional hazard regression analysis. Statistical significance was set at *p* < 0.05. Analysis was performed using SAS Enterprise Guide software (version 7.1; SAS Institute, Cary, NC, USA) and STATA version 17.0 (StataCorp, College Station, TX, USA).

## 3. Results

### 3.1. Baseline Characteristics of Study Participants

Table 1 presents the characteristics of the study participants in the baseline year (2008–2009) and after evaluating lifestyle factors over an 8-year period. After excluding those with missing responses, the number of study participants reached 14,721. Participants were South Korean men ≥ 65 years, with a mean age of 70.4 ± 3.9 years. Approximately half (47.1%) of the population were grouped into the 65–69 age group. Participants with a BMI ≥ 25 were defined as obese and accounted for 30.5% of the study population. In total, 25.7% of participants were current smokers, 25.7% were current drinkers, and 74.2% did not engage in regular physical activity. Of the three comorbidities that were considered (diabetes, hypertension, and dyslipidemia), hypertension had the highest prevalence. Of all participants, 53.2% were diagnosed with hypertension, 19.5% were diagnosed with dyslipidemia, and 17.5% were diagnosed with diabetes. The distributions of the characteristics were similar after exclusion.

### 3.2. Risk of Cancer According to Lifestyle Factors in Older Korean Men

During the mean follow-up of 8.04 years, the CIR for cancer was 2.10. Table 2 presents the CIRs, HRs, and 95% CIs for cancer according to the sociodemographic characteristics and lifestyle factors of older Korean men. Multivariate analysis showed that the HRs (95% CI) for men with cancer aged 70–74 and ≥75 years were 1.26 (1.22–1.31) and 1.48 (1.41–1.56), respectively, compared to those aged 65–69. The HRs (95% CI) for cancer for current smokers, current drinkers, and those not engaged in regular physical activity were 1.38 (1.33–1.43), 1.15 (1.10–1.19), and 1.06 (1.02–1.10), respectively, compared to non-smokers, non-drinkers, and those who engaged in regular physical activity. Compared to participants without comorbidities, the HRs (95% CI) for cancer of those diagnosed with diabetes and hypertension were 1.10 (1.05–1.15) and 1.05 (1.01–1.09), respectively.

### 3.3. Association Between the Number of Poor Lifestyle Factors and Cancer

Table 3 lists the risk of cancer according to the number of lifestyle risk factors in older Korean men at a single point. Compared to participants without a poor lifestyle risk factor, the HRs were 1.09 (1.04–1.15), 1.39 (1.31–1.47), and 1.63 (1.52–1.75) for those with one, two, and three poor lifestyle factors, respectively. The risk of cancer increased as the lifestyle risk factor score increased (*p* for trend < 0.001).

### 3.4. Association Between Lifestyle Risk Score and Cancer

Table 4 shows the risk of cancer in older Korean men according to lifestyle risk score over 8 years (2002–2009). Compared to those with the lowest lifestyle risk score (0–2), the HRs for scores of 3–5, 6–8, and 9–12 were 1.10 (0.98–1.23), 1.54 (1.37–1.73), and 1.72 (1.48–1.99), respectively. The risk of cancer increased with increasing lifestyle risk score over 8 years in older Korean men (*p* for trend < 0.001).

## 4. Discussion

The risk of cancer increased with lifestyle risk score over 8 years, and the number of poor lifestyle factors increased in older Korean men. Many studies have been conducted on the association between unhealthy lifestyle factors and the risk of cancer; however, this is the first study that has collected data on 8 years’ worth of lifestyle habits and collated them into lifestyle risk scores to investigate the relationship between unhealthy lifestyle habits and cancer. Lifestyle factors were tracked over four phases (2002–2003, 2004–2005, 2006–2007, and 2008–2009), and cancer incidence was tracked from 1 January 2010 to 31 December 2019.

The association between lifestyle risk factors and the duration (person years) of cancer was examined. The CIR for cancer was 2.1 for older Korean men. In 2024, the United States and South Korea are predicted to have 2,001,140 and 292,221 new cases of cancer, respectively [7,26]. According to statistics from the National Institute of Health, cancer incidence exceeds 1000 per 100,000 people in age groups ≥60 years [27]. Globally, nearly 60% of cases diagnosed as cancer occur among individuals aged ≥65 years [28].

Practicing unhealthy lifestyle habits (smoking, drinking, and physical inactivity) led to a higher risk of cancer in older Korean men. Such findings can be explained by physiological damage engendered by each unhealthy lifestyle habit resulting in human carcinogenesis. For instance, cigarette smoke comprises >7000 chemical compounds, many of which (such as 1, 3-butadiene, acetaldehyde, N-nitrosamine, and polycyclic aromatic hydrocarbons) are considered carcinogenic to humans by the International Agency for Research on Cancer [29]. These chemicals result in DNA adducts that cause permanent mutation in critical regions, including tumor suppressor gene *TP53* and oncogene *KRAS* [29]. Moreover, cigarette smoke causes oxidative damage, triggers inflammation, and promotes gene methylation, all of which contribute to the development of cancer [29]. Similarly, alcohol causes chronic tissue inflammation, affects sex hormone levels, reduces folate concentration, increases DNA methylation, and causes cirrhosis, leading to various types of cancer [30]. In addition, physical inactivity is known to potentially promote carcinogenesis; the most mentioned biochemical mechanisms include enhancement of immune function, stimulation of apoptosis, upregulation of tumor suppressors, the release of myokines, and the prevention of non-communicable diseases [15,31].

Some studies have examined the combined effects of unhealthy lifestyle habits on cancer [32,33,34,35]. In general, these studies reported a reduced burden of cancer in the absence of combined risk factors. In addition, these lifestyle habits demonstrated a cumulative effect when observed repetitively over multiple phases. For instance, in the case of drinking, individuals with moderate or heavy alcohol consumption who stopped drinking initially showed a higher risk of cancer; however, this elevated risk diminished when abstinence was maintained over time [36]. To confirm the cumulative effect of the three unhealthy lifestyle habits, this study measured the lifestyle risk scores at four phases over 8 years and analyzed their association with cancer incidence. Considering that one-quarter of older Korean men consume alcohol and currently smoke, and over 74% do not engage in regular physical activity, highlighting the effects of unhealthy lifestyle behaviors is expected to reduce the incidence of cancer among older Korean men.

Additional factors relating to cancer include age, diabetes, and hypertension. The hallmarks of aging include chronic inflammation, genomic instability, telomere attrition, and stem cell exhaustion, which all affect carcinogenesis [37]. However, diabetes was suggested to be related to only certain types of cancers, including gastric cancer [38]. In some studies, diabetes showed no significant relationship to lung and pancreatic cancers in men and even showed a protective effect in prostate cancer [39,40,41]. Similarly, hypertension is suggested to provide a protective effect against some cancers, including colorectal cancer [42]. However, hypertension is associated with certain cancers and is known to be the most common cardiovascular comorbidity reported in patients with cancer [43]. The relationship between diabetes, hypertension, and cancer may also be related to age, as both diseases commonly occur due to aging.

This study suggested no significant relationship between a high BMI, dyslipidemia, and an increasing incidence and risk of cancer. The lack of association between BMI and cancer may be due to BMI being an inadequate measure of adiposity, especially in older adults. In this study, BMI was used as the primary indicator of obesity, as it was assessed during the data collection stage of the NHIS-Senior Cohort. However, as BMI only accounts for one’s height and weight, it does not consider changes in body composition due to aging. Notably, among older adults, those with a slightly higher BMI may be considered healthier than those with a lower index, as a higher BMI is often linked to higher muscle mass. Therefore, the increased adiposity and decreased muscle mass of older adults were not considered, suggesting diagnostic inaccuracy in identifying obesity [44]. The lack of association between dyslipidemia and cancer may be due to this study covering all other causes of cancer and not just those related to obesity. The types of cancers proposed to hold an association with dyslipidemia in men are limited, such as gastric, pancreatic, colon, endometrial, esophageal, prostate, and hematologic cancers [45]. As this study utilized an operational diagnosis and not a direct measurement of cholesterol levels, the study participants included patients with well-controlled dyslipidemia. This may have also affected dyslipidemia prohibiting the induction of a noticeable effect on cancer, which is a result that does not align with the known relationship between dyslipidemia and certain cancers [45].

This study has a few limitations. First, there is a possibility of inaccurate ICD-10 codes being recorded for patients. Second, due to the changes in the national health check-up, the definition of regular physical activity in 2009 is different from that in 2002 and 2008. Third, macro-individual factors that may be relevant to cancer such as urban-rural location or exposure to pollutants were not considered. Fourth, as we measured lifestyle factors four times over an 8-year period, a large number of missing cases developed; however, the characteristics of the study participants at each stage of exclusion were similar (Table S1). Fifth, earlier or later exposures, and more or less intense exposures to lifestyle risk factors may have influenced cancer differently. Such considerations may be developed in our future research. Despite such potential limitations, this study used a nationwide cohort that well represents the older Korean population. Furthermore, this study measured lifestyle factors four times over an 8-year period to evaluate the cumulative effects of multiple lifestyle factors on cancer.

## 5. Conclusions

In our study, the cumulative burden of 8 years of lifestyle risk factors was analyzed, particularly its association with the incidence of cancer among older South Korean men. The risk of cancer increased as the cumulative lifestyle risk score increased. Therefore, practicing and maintaining healthy lifestyles is crucial to preventing cancer in older Korean men.

## Figures and Tables

**Table 1:** Characteristics of the study participants in the baseline year (2008–2009) and after evaluating lifestyle factors over an 8-year period.

	Baseline Year (2008–2009) (N = 64,756)	After Exclusion (N = 14,721)
	N (%)	N (%)
Age (years)		
65–69	30,514 (47.1)	6313 (42.9)
70–74	23,311 (36.0)	5664 (38.5)
≥75	10,931 (16.9)	2744 (18.6)
Mean age (SD)	70.4 (3.9)	70.7 (4.0)
Insurance premium		
1st quartile	14,264 (22.0)	3385 (23.0)
2nd quartile	19,544 (30.2)	4451 (30.2)
3rd quartile	19,580 (30.2)	4162 (28.3)
4th quartile	11,368 (17.6)	2723 (18.5)
Body mass index (kg/m^2^)		
<18.5	2574 (4.0)	527 (3.6)
18.5–22.9	24,779 (38.3)	5632 (38.3)
23.0–24.9	17,681 (27.3)	4221 (28.7)
≥25	19,722 (30.5)	4341 (29.5)
Smoking status		
Non-smoker	48,117 (74.3)	11,493 (78.1)
Current smoker	16,639 (25.7)	3228 (21.9)
Alcohol consumption		
Non-drinker	48,134 (74.3)	11,058 (75.1)
Drinker	16,622 (25.7)	3663 (24.9)
Regular physical activity		
No	48,066 (74.2)	10,729 (72.9)
Yes	16,690 (25.8)	3992 (27.1)
Comorbidities		
Diabetes	11,344 (17.5)	2372 (16.1)
Hypertension	34,472 (53.2)	8003 (54.4)
Dyslipidemia	12,637 (19.5)	2894 (19.7)

SD: standard deviation.

**Table 2:** Incidence and risk of cancer according to lifestyle factors in older Korean men (N = 64,756).

	N	Event (N)	Duration (Person Years)	CIR	Unadjusted HR (95% CI)	* Adjusted HR (95% CI)
Age (years)						
65–69	30,514	5715	3,114,048	1.84	1.00	1.00
70–74	23,311	5008	2,208,876	2.27	1.24 (1.19–1.29)	1.26 (1.22–1.31)
≥75	10,931	2407	924,742	2.60	1.43 (1.36–1.50)	1.48 (1.41–1.56)
Insurance premium						
1st quartile	14,264	2796	1,386,566	2.02	1.00	1.00
2nd quartile	19,544	3983	1,878,201	2.12	1.05 (1.00–1.10)	1.04 (0.99–1.09)
3rd quartile	19,580	3999	1,882,989	2.12	1.05 (1.00–1.11)	1.03 (0.98–1.08)
4th quartile	11,368	2352	1,099,911	2.14	1.06 (1.00–1.12)	1.03 (0.98–1.09)
BMI (kg/m^2^)						
<18.5	2574	504	211,583	2.38	1.10 (1.01–1.21)	1.04 (0.95–1.14)
18.5–22.9	24,779	5076	2,337,538	2.17	1.00	1.00
23.0–24.9	17,681	3557	1,738,471	2.05	0.94 (0.90–0.98)	0.98 (0.93–1.02)
≥25	19,722	3993	1,960,075	2.04	0.94 (0.90–0.98)	0.99 (0.95–1.03)
Smoking status						
Non-smoker	48,117	9076	4,709,004	1.93	1.00	1.00
Current smoker	16,639	4054	1,538,663	2.63	1.37 (1.32–1.42)	1.38 (1.33–1.43)
Alcohol consumption						
Non-drinker	48,134	9369	4,640,642	2.02	1.00	1.00
Drinker	16,622	3761	1,607,025	2.34	1.16 (1.12–1.20)	1.15 (1.10–1.19)
Regular physical activity						
Yes	16,690	3280	1,659,927	1.98	1.00	1.00
No	48,066	9850	4,587,740	2.15	1.09 (1.05–1.13)	1.06 (1.02–1.10)
Comorbidities						
Diabetes	11,344	2318	1,039,458	2.23	1.08 (1.03–1.13)	1.10 (1.05–1.15)
Hypertension	34,472	7015	3,273,590	2.14	1.04 (1.01–1.08)	1.05 (1.01–1.09)
Dyslipidemia	12,637	2514	1,222,225	2.06	0.97 (0.93–1.02)	0.98 (0.93–1.02)

CIR: cumulative incidence rate, HR: hazard ratio, CI: confidence interval, BMI: body mass index. * Adjusted for age, insurance premium, BMI, smoking status, alcohol consumption, regular physical activity, diabetes, hypertension, and dyslipidemia.

**Table 3:** Risk of cancer according to the number of poor lifestyle factors at the baseline year in older Korean men (N = 64,756).

Number of Poor Lifestyle Factors	N	Event (N)	Unadjusted HR (95% CI)	* Adjusted HR (95% CI)
0	10,161	1831	1.00	1.00
1	32,667	6162	1.09 (1.03–1.15)	1.09 (1.04–1.15)
2	17,124	3908	1.35 (1.27–1.42)	1.39 (1.31–1.47)
3	4804	1229	1.55 (1.45–1.67)	1.63 (1.52–1.75)
*p* for trend			<0.001	<0.001

HR: hazard ratio, CI: confidence interval * Adjusted for age, insurance premium, BMI, diabetes, hypertension, and dyslipidemia.

**Table 4:** Risk of cancer according to lifestyle risk score over 8 years in older Korean men (N = 14,721).

Lifestyle Risk Score	N	Event (N)	Unadjusted HR (95% CI)	* Adjusted HR (95% CI)
0–2	2550	435	1.00	1.00
3–5	7001	1260	1.08 (0.96–1.20)	1.10 (0.98–1.23)
6–8	3938	920	1.47 (1.31–1.65)	1.54 (1.37–1.73)
9–12	1232	305	1.58 (1.37–1.83)	1.72 (1.48–1.99)
*p* for trend			<0.001	<0.001

HR: hazard ratio, CI: confidence interval. * Adjusted for age, insurance premium, BMI, diabetes, hypertension, and dyslipidemia.

## Data Availability

Restrictions apply to the availability of these data. Data were obtained from National Health Insurance Sharing Service (NHISS) and are available from [http://nhiss.nhis.or.kr/bd/ab/bdaba021eng.do] (accessed on 1 January 2025) with the permission of the National Health Insurance Service.

## Supplementary Materials

The following supporting information can be downloaded at: https://www.mdpi.com/article/10.3390/cancers17030426/s1, Table S1: Characteristics of the study participants after excluding missing responses for lifestyle factors.
