# Supplementary material for: Cumulative Burden of Lifestyle Risk Factors on Cancer in Older Korean Men: A Nationwide Retrospective Cohort Study
Source: Cancers (Basel). 2025 Jan 27;17(3):426. doi: 10.3390/cancers17030426 (PMC11815911; doi:10.3390/cancers17030426)
Supplement: Supplementary file 1 [file cancers-17-00426-s001.zip › cancers-3432597-supplementary.pdf]

Supplementary Table S1. Characteristics of the study participants after excluding missing responses for lifestyle factors

Measurement period for lifestyle factors	2008 – 2009	2008 – 2009<br>2006 – 2007	2008 – 2009<br>2006 – 2007<br>2004 - 2005	2008 – 2009<br>2006 – 2007<br>2004 – 2005<br>2002 – 2003
	Men<br>(N = 64756)	Men<br>(N = 38927)	Men<br>(N = 24095)	Men<br>(N = 14721)
	N (%)	N (%)	N (%)	N (%)
Age (years)				
65 – 69	30514 (47.1)	16311 (41.9)	10138 (42.1)	6313 (42.9)
70 – 74	23311 (36.0)	15520 (39.9)	9544 (39.6)	5664 (38.5)
≥ 75	10931 (16.9)	7096 (18.2)	4413 (18.3)	2744 (18.6)
Mean age (SD)	70.4 (3.9)	70.7 (3.9)	70.7 (3.9)	70.7 (4.0)
Insurance premium				
1st quartile	14264 (22.0)	8372 (21.5)	5360 (22.2)	3385 (23.0)
2nd quartile	19544 (30.2)	11579 (29.7)	7209 (29.9)	4451 (30.2)
3rd quartile	19580 (30.2)	11921 (30.6)	7118 (29.5)	4162 (28.3)
4th quartile	11368 (17.6)	7055 (18.1)	4408 (18.3)	2723 (18.5)
Body mass index (kg/m^2)				
< 18.5	2574 (4.0)	1506 (3.9)	878 (3.6)	527 (3.6)
18.5 – 22.9	24779 (38.3)	14966 (38.4)	9233 (38.3)	5632 (38.3)
23.0 – 24.9	17681 (27.3)	10813 (27.8)	6846 (28.4)	4221 (28.7)
≥ 25	19722 (30.5)	11642 (29.9)	7138 (29.6)	4341 (29.5)
Smoking status				
Non-smoker	48117 (74.3)	29572 (76.0)	18651 (77.4)	11493 (78.1)
Current smoker	16639 (25.7)	9355 (24.0)	5444 (22.6)	3228 (21.9)
Alcohol consumption				
Non-drinker	48134 (74.3)	29316 (75.3)	18227 (75.6)	11058 (75.1)
Drinker	16622 (25.7)	9611 (24.7)	5868 (24.4)	3663 (24.9)
Regular physical activity				
No	48066 (74.2)	28665 (73.6)	17563 (72.9)	10729 (72.9)
Yes	16690 (25.8)	10262 (26.4)	6532 (27.1)	3992 (27.1)
Comorbidities				
Diabetes	11344 (17.5)	6531 (16.8)	3987 (16.5)	2372 (16.1)
Hypertension	34472 (53.2)	20890 (53.7)	13059 (54.2)	8003 (54.4)
Dyslipidemia	12637 (19.5)	7556 (19.4)	4725 (19.6)	2894 (19.7)

SD: Standard deviation
